# Nutritional balance of essential amino acids and carbohydrates of the adult worker honeybee depends on age

**DOI:** 10.1007/s00726-014-1706-2

**Published:** 2014-03-13

**Authors:** Pier P. Paoli, Dion Donley, Daniel Stabler, Anumodh Saseendranath, Susan W. Nicolson, Stephen J. Simpson, Geraldine A. Wright

**Affiliations:** 1 Centre for Behaviour and Evolution, Institute of Neuroscience, Newcastle University, Newcastle upon Tyne, NE1 7RU UK; 2 School of Agriculture, Food and Rural Development, Newcastle University, Newcastle upon Tyne, NE1 7RU UK; 3 Department of Zoology and Entomology, University of Pretoria, Private Bag X20, Hatfield, Pretoria, 0002 South Africa; 4 Charles Perkins Centre, University of Sydney, Sydney, NSW 2006 Australia

**Keywords:** Honeybee, Amino acid, Nutrition, Protein-to-carbohydrate ratio, *Apis mellifera*, Diet

## Abstract

Dietary sources of essential amino acids (EAAs) are used for growth, somatic maintenance and reproduction. Eusocial insect workers such as honeybees are sterile, and unlike other animals, their nutritional needs should be largely dictated by somatic demands that arise from their role within the colony. Here, we investigated the extent to which the dietary requirements of adult worker honeybees for EAAs and carbohydrates are affected by behavioural caste using the Geometric Framework for nutrition. The nutritional optimum, or intake target (IT), was determined by confining cohorts of 20 young bees or foragers to liquid diets composed of specific proportions of EAAs and sucrose. The IT of young, queenless bees shifted from a proportion of EAAs-to-carbohydrates (EAA:C) of 1:50 towards 1:75 over a 2-week period, accompanied by a reduced lifespan on diets high in EAAs. Foragers required a diet high in carbohydrates (1:250) and also had low survival on diets high in EAA. Workers exposed to queen mandibular pheromone lived longer on diets high in EAA, even when those diets contained 5× their dietary requirements. Our data show that worker honeybees prioritize their intake of carbohydrates over dietary EAAs, even when overeating EAAs to obtain sufficient carbohydrates results in a shorter lifespan. Thus, our data demonstrate that even when young bees are not nursing brood and foragers are not flying, their nutritional needs shift towards a diet largely composed of carbohydrates when they make the transition from within-hive duties to foraging.

**Electronic supplementary material:**

The online version of this article (doi:10.1007/s00726-014-1706-2) contains supplementary material, which is available to authorized users.

## Introduction

All animals require a dietary source of essential amino acids (EAAs) which are used for growth, somatic maintenance, and reproduction. EAAs are obtained by consuming the protein found in other animals or plants and are in greatest demand during periods of growth early in life (Behmer 2009; Tigreros 2013). In contrast to juvenile animals, adults mainly require amino acids for basic somatic functions (e.g. production of enzymes, peptide or amine signalling, tissue repair, immune function) or reproduction, and their needs for EAA decline with age (Millward et al. 1997; van de Rest et al. 2013). Reproduction, in the form of allocation of resources to eggs or offspring by females (O’Brien et al. 2002) or the donation of nuptial gifts and the production of sperm by males (Voigt et al. 2008), also places demands on the acquisition of amino acids in adult diets, which is often manifested as a trade-off between lifespan and protein/amino acid ingestion (Fanson et al. 2012; Grandison et al. 2009; Maklakov et al. 2008).

Adult eusocial insects such as ants and bees live in colonies of closely related, largely sterile adults that engage in a division of labour that includes caring for the queen and brood. In honeybees, behavioural caste correlates with age within the colony. After eclosion, adult worker bees perform within-colony activities such as cleaning, food storage, rearing larvae, and attending the queen (Winston 1987). These bees continue to perform within-hive behavioural tasks as a function of their exposure to the queen’s mandibular pheromone (QMP); bees near the queen remain more ‘nurse-like’ whereas bees exposed to less of the queen’s pheromones become foragers (Pankiw et al. 1998). Honeybees are unique, even among social insects, because nurses provision larvae, other workers, and the queen with glandular secretions as food––royal jelly––which is the main source of protein that larvae receive for the first three instars after hatching (Winston 1987). Because most adult bees or ants are sterile, their nutritional requirements for amino acids should be considerably less than those of queens or drones. However, the production of royal jelly is likely to place metabolic demands on young bees (e.g. nurses) for dietary amino acids (Crailsheim 1990): how much that demand exceeds their own requirements for somatic maintenance is unknown. As young bees mature and pass through their behavioural ontogeny, they stop eating pollen and lose the ability to digest solid proteins (Crailsheim 1986, 1990; Lass and Crailsheim 1996; Moritz and Crailsheim 1987); they also stop producing glandular secretions and tending larvae and start building wax comb, packing food into the cells, guarding the colony entrance and eventually become foragers (Winston 1987). Furthermore, during this period, their physiology changes substantially: their mandibular glands and ovaries atrophy (Winston 1987) and their fat body reduces (Seehuus et al. 2007; Ament et al. 2008). How much their nutritional optimum depends on their behavioural caste and age has not yet been tested.

The Geometric Framework (GF) for nutrition is a modelling method developed to identify an animal’s optimal intake of key nutrients such as protein and carbohydrate, and the regulatory priorities for different nutrients and performance consequences when animals are confined to suboptimal diets (Simpson and Raubenheimer 1993, 2012). The GF is based on the principle that animals require multiple nutrients simultaneously. The requirement to achieve the optimal proportion of nutrients in their diet forces them to consume a varied diet and/or make trade-offs by overeating or undereating specific nutrients in available foods (Raubenheimer and Simpson 1997). When animals are restricted to a diet containing a set proportion of nutrients, the amount they eat should reflect a ‘rule of compromise’ that is governed by homeostatic mechanisms tuned to regulate feeding behaviour (Raubenheimer and Simpson 1997; Simpson et al. 2004). These rules are dictated both by nutrient requirements, but also by costs associated with over or under-consuming nutrients relative to requirements (Simpson et al. 2004). The optimal amounts and ratio of specific nutrients, or the ‘intake target’ (IT), for an animal can be identified in various ways, including by examining the food intake of animals when they are confined to one of several foods composed of different proportions of two or more nutrients (Simpson et al. 2004) or by offering different pairwise choices of nutritionally complementary foods (Chambers et al. 1995).

Here, we tested how caste determined the adult worker honeybee’s nutritional requirements for EAAs using the principles in the GF (Simpson and Raubenheimer 1993; Simpson et al. 2004). By confining cohorts of workers to single diets composed of specific proportions of sucrose and the ten EAAs required by honeybees (i.e. protein), we identified the ITs of young bees (days 0–14 from emergence within the colony) and foragers (collected from outside the colony). Diet was limited to carbohydrates (sucrose) and amino acids because these are the main components of honey (Anklam 1998; Hermosin et al. 2003): one of the foods eaten by young workers within the colony and the only food consumed by foragers (with the exception of glandular secretions received during trophallaxis, see Crailsheim 1998). To identify costs associated with over consumption of specific nutrients, we also measured how dietary intake of EAAs influenced survival. To confirm that the costs associated with overeating EAAs was a result of physiological changes associated with age and behavioural caste, we exposed newly enclosed workers to synthetic QMP and measured food consumption and survival on diets high in EAA. This study is the first to show that the nutritional requirements of honeybees change as a function of age and behavioural role, and has implications for dietary intervention strategies designed to improve honeybee health.

## Materials and methods

### Animals

Frames of newly emerged workers were removed from two colonies of *Apis mellifera* “Buckfast” hybrid honeybees kept in outdoor colonies at Newcastle University. Each frame was placed in a box in a controlled temperature room at 34 °C and 60 % relative humidity. Newly emerged bees were brushed off the frame each day; foraging workers were collected daily at the hive entrance by capturing individuals in plastic sample tubes. For each cohort, 20 bees were placed in a Perspex box (11 × 6 × 20 cm) with five feeding tubes consisting of 2-ml microcentrifuge tubes (four 3-mm holes were drilled along the top of each tube). Four feeding tubes were filled with a treatment solution; each box also had a water tube. The boxes were placed in a constant temperature room at 34 °C and 60 % RH. Experiments continued for 14 days for newly emerged workers (i.e. nurse bees), and 7 days for foraging workers (or as long as the bees survived in both cases). The number of dead bees in each box was counted daily.

### Diets

Each of the ten EAAs needed by honeybees (de Groot 1953; methionine, tryptophan, arginine, lysine, histidine, phenylalanine, isoleucine, threonine, leucine, valine) was added to a 1.0 M sucrose solution (Table S1). Ratios of amino acids to carbohydrates (sucrose) were calculated on a molar–molar basis as the following: 1:750, 1:500, 1:250, 1:100, 1:75, 1:50, 1:10, 1:5. All ten amino acids were was added at the same concentration: for example, for the 1:10 diet, the total final concentration of the amino acids was 0.1 M, with each amino acid present at a concentration of 0.01 M.

To measure food consumption, each tube containing nutrient solution or water was weighed, placed in the box, and then reweighed 24 h later. Each tube was replaced with a new, full tube daily. The difference in weight was a measure of the amount consumed in a 24-h period. A control for the evaporation rate of the solution from each tube was performed for each diet by monitoring weight loss in feeding tubes in empty boxes daily for a 5-day period. The final figure for total consumption from each tube was adjusted for evaporation rate by subtracting the amount of solution evaporated from the control boxes with no bees; changes in concentration were also estimated (Table S2). Total daily consumption represented the sum of the adjusted weight of all four feeding tubes; this figure was then divided by the number of live bees remaining on that day. Total carbohydrate and amino acid consumption was calculated by multiplying the amount consumed per bee by the weight of sucrose and the weight of amino acid in 1 ml of solution.

For the sucrose only experiments, cohorts of 20 bees were confined to feeding on 1.0 M sucrose solution for 14 days for young bees and 7 days for foragers. The total volume of solution was measured each day. The volume was adjusted by the evaporation rate in each tube as above.

### Queen mandibular pheromone

Cohorts of 20 newly emerged honeybees were kept in the same conditions as described previously. QMP was administered to the treatment group by placing a 2-cm strip of BeeBoost QMP substitute (Pherotech) in each box. Honeybees without QMP were maintained in a separate incubator to avoid contamination with QMP, as were the diets. The diets were administered as described previously; the two diets chosen were sucrose only and the 1:5 diet. Experiments continued for 14 days, with survival and consumption recorded each day for each box (10 per treatment).

### Statistics

Daily and total consumption data were analysed using generalized linear models or repeated-measures ANOVA using SPSS (IBM SPSS Statistics v.19) with diet as a main effect. *Post hoc* comparisons were made using least squares difference (LSD) analysis with significance at *P* ≤ 0.05. The impact of diet on survival was analysed using a Cox regression (Coxreg) analysis to calculate the hazard ratio (HR) or by Kaplan–Meier analysis. Diets were compared per day over the experimental period using log-rank, pairwise comparisons for each strata, and LSD post hoc tests.

## Results

### Nutritional intake target of worker bees shifts towards carbohydrate with age and caste

**Fig. 1:**
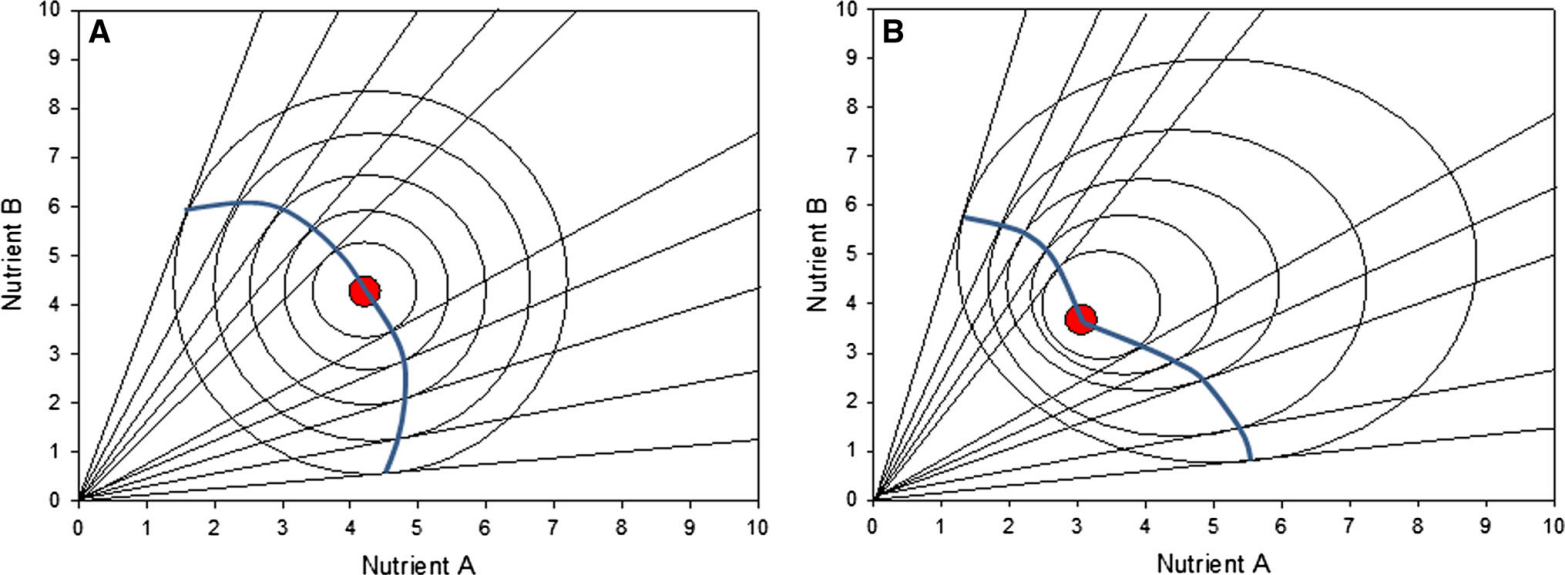
Model of dietary regulation when animals are confined to diets of specific proportions of two macronutrients. When unable to reach an optimal diet composition (the IT, indicated as *red dot*) they must trade-off overeating the nutrient in excess against undereating the nutrient in deficit. Across an array of diets, these trade-off points form an intake array, the shape of which reflects the underlying fitness landscape describing the costs of ingesting nutrient excess and deficits. Fig. 1a, b showcases whether the costs of eating excesses are less than the costs of ingesting deficits relative to the IT. Figure 1a, b indicates cases for quadratic cost functions with these being symmetrical for the two nutrients in **a** and asymmetrical in **b**. Note how in **b** the array hinges outwards at the IT, and hence the hinge point in an array of this form can be used to infer the position of the IT (colour figure online)

The GF model described by Simpson and Raubenheimer (1993, 2012) predicts that animals make compromises when they are confined to unbalanced diets that reflect the fitness costs associated with over-ingesting and under-ingesting specific nutrients relative to the IT (Fig. 1a, b). In Fig. 1a, the case is illustrated where the underlying fitness landscape is symmetrical about the IT, i.e. the fitness costs of over-ingestion equal the costs of under-ingestion for a given nutrient relative to the IT (and in the case illustrated in Fig. 1a, the costs are also symmetrical between the two nutrients). Here, the intake array across an array of unbalanced food rails forms a smooth arc (Simpson et al. 2004). In Fig. 1b, by contrast, the costs of ingesting excesses are less than those of ingesting deficits of either or both nutrients (Simpson et al. 2004). In this case, the intake array across a range of food rails in not a smooth arc, but rather it can be inferred that the IT lies at the hinge point of the array––identified as a red dot in Fig. 1.

**Fig. 2:**
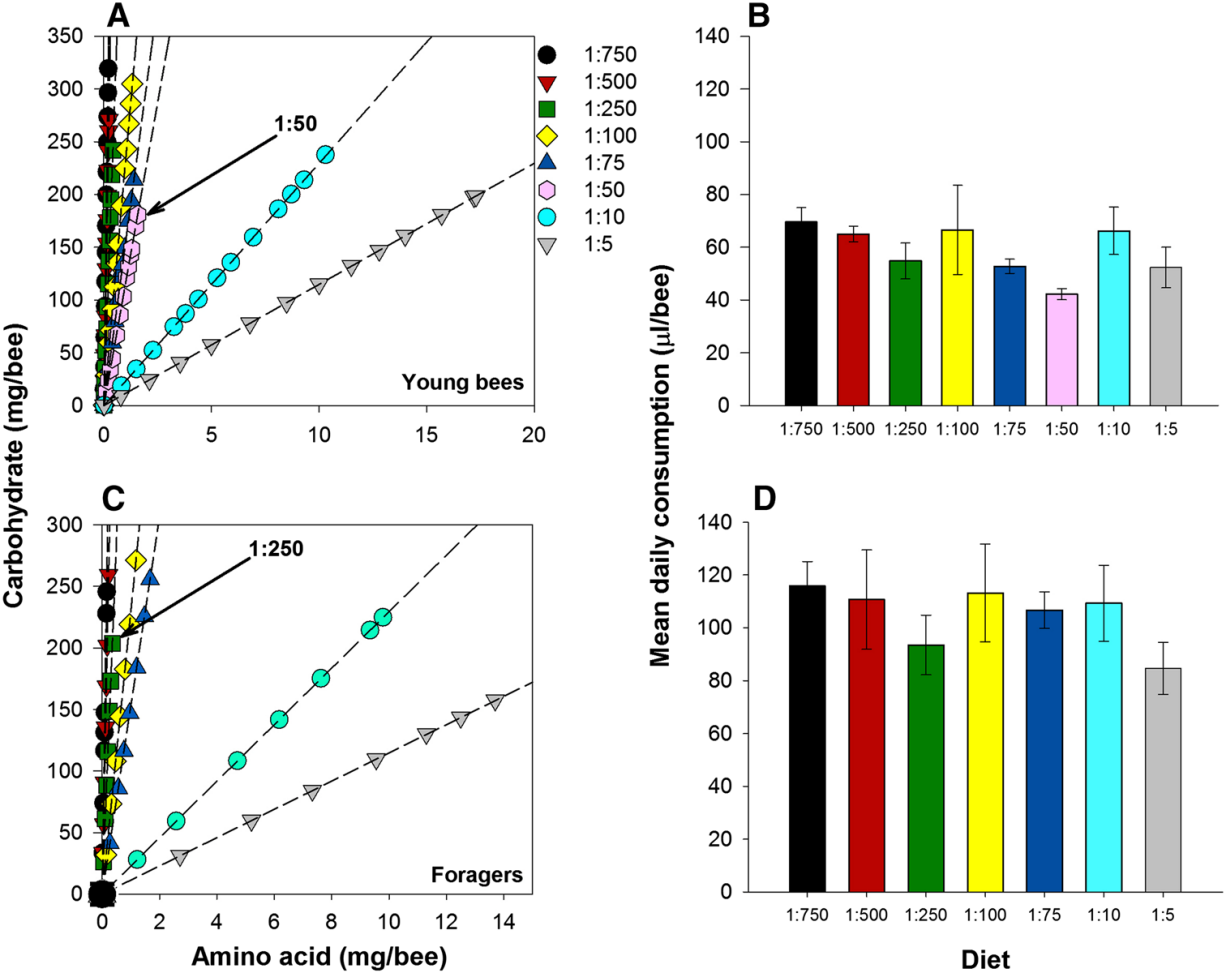
Nutritional regulation in young honeybees and foragers. The demand for EAAs decreases when worker honeybees become foragers (*n* = 10 cohorts of 20 bees each per rail). **a** Newly emerged workers confined to diets of specific proportions of EAAs and sucrose regulated their intake around an IT of 1:50. **b** The total volume of diet solution consumed by young bees depended on the proportion of EAAs and carbohydrates in the diet (one-way ANOVA, 1:50 × all other diets, all *P* < 0.033). **c** Foragers use an asymmetric rule of compromise when regulating their intake, such that intake is always skewed towards carbohydrates; the IT was estimated to 1:250. **d** The average daily volume consumed by foragers did not depend on diet

**Fig. 3:**
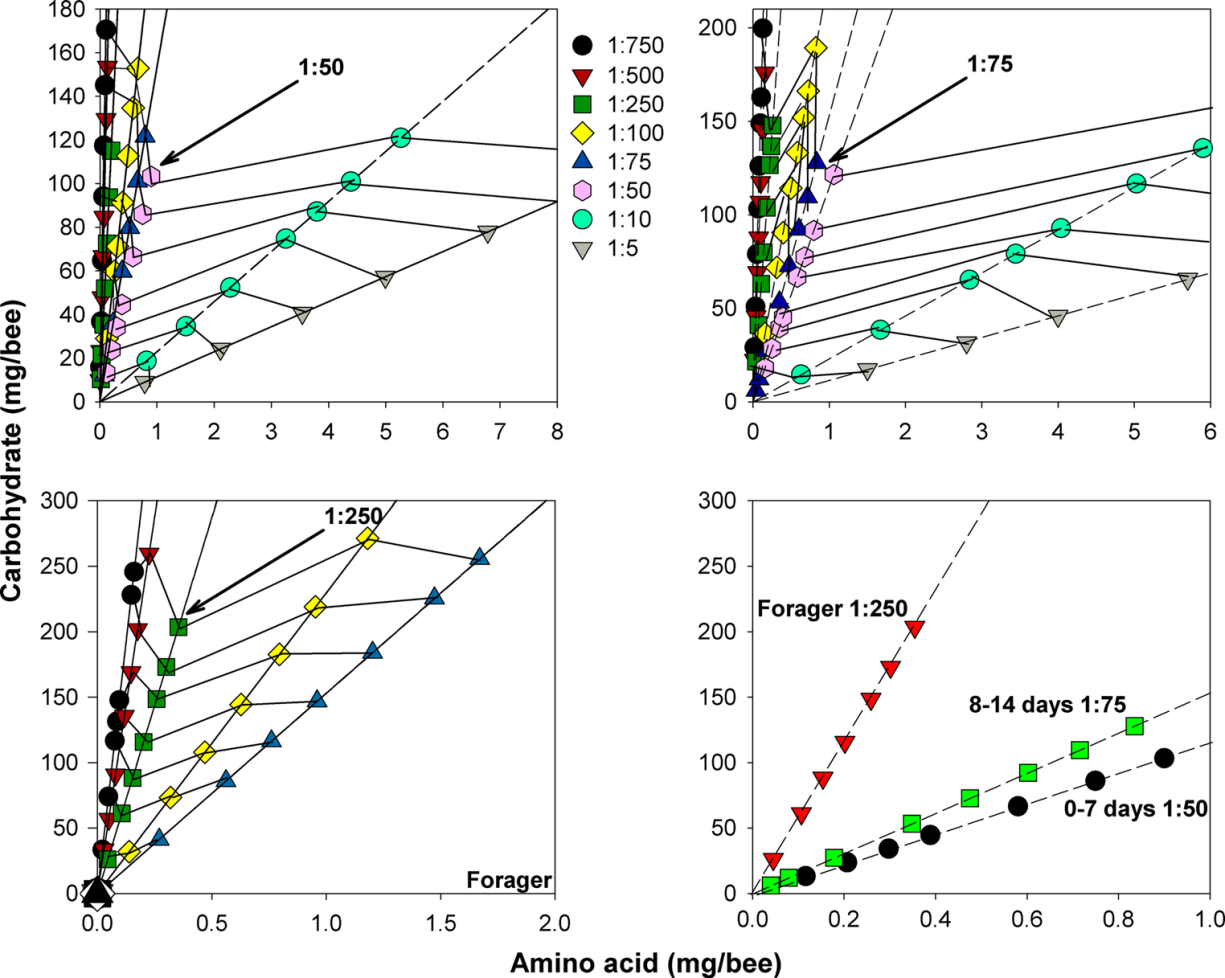
ITs of honeybees shift towards carbohydrates as bees age. The IT of newly emerged bees **a**, young worker bees **b**, and foragers **c** is re-plotted from a subset of data in Fig. 2. **d** The trajectory for bees fed the diet closest to the IT of all three age groups is shown; foragers require ~5-fold less dietary EAAs than young bees

By confining bees to specific proportions of EAAs and sucrose, we were able to identify the IT and the rule of compromise for diets composed of EAAs and carbohydrates (EAA:C). The IT of queenless, newly emerged (young) worker honeybees shifted towards carbohydrates as the workers aged and transitioned to the forager caste (Figs. 2, 3). The intake arrays recorded in the present experiment tended to hinge at a point of least consumption on one diet and open outwards as bees ingested more on diets diverging from this ratio, indicating that the bees followed an asymmetrical quadratic rule of compromise as in Fig. 1b (Simpson et al. 2004).

Young bees consistently overate diets high in EAAs to obtain sufficient carbohydrate (Fig. 2a, GLZM, main effect, diet: *χ*_6_^2^ = 182, *P* < 0.001), but would also overeat diets containing a high proportion of carbohydrates to obtain sufficient EAAs (GLZM, main effect, diet: *χ*_6_^2^ = 182, *P* < 0.001). The hinge point in this graph, when compared to Fig. 1, indicates that young, adult worker honeybees have an IT of 1:50 (Compared to previous work using protein in diet instead of amino acids, our 1:50 diet is equivalent to a weight-for-weight diet of 1:115, amino acid-to-carbohydrate, Table S1). The daily average volume of each diet solution consumed by the cohorts of young bees was not significantly different (Fig. 2b, GLZM, main effect, diet: *χ*_7_^2^ = 10.8, *P* = 0.145).

Foragers died rapidly when confined to cages within the lab; for this reason, we evaluated the survival and consumption of foragers over 7 days rather than 14 days. Using the same logic as above in Fig. 1, we estimate that the IT of foragers was 1:250 (Fig. 2c). The foragers defended their intake of carbohydrates at the expense of overeating EAAs: the cumulative quantity of EAAs consumed depended on the diet (Fig. 2c, GLZM, main effect, diet: *χ*_6_^2^ = 182, *P* < 0.001), but the amount of carbohydrates was constant (GLZM, main effect, diet: *χ*_6_^2^ = 10.7, *P* = 0.097). Again, the daily average volume of each diet solution consumed was not significantly different (Fig. 2d, GLZM, main effect, diet: *χ*_6_^2^ = 4.83, *P* = 0.565).

The shift towards a diet high in carbohydrates between young bees and foragers is most obvious when a subgroup of the rails around the IT for newly emerged bees, young bees, and foragers is plotted separately (Fig. 3). Since we noticed a large change in the ability to survive on diets high in EAAs, we compared whether there was an effect of diet on the IT of nurses aged 0–7 days (Fig. 3a), nurses aged 8–14 days (Fig. 3b), and foragers (Fig. 3c). The amount of diet consumed per day depended on the age group and the dietary ratio of EAA:C (three-way interaction: repeated-measures ANOVA, day × age × diet*, F*_8,702_ = 6.44, *P* < 0.001). Young bees during days 0–7 had an IT closest to 1:50, whereas bees aged 8–14 days had an IT that had shifted towards 1:75. When the ITs of all three groups are compared (Fig. 3d), it is clear that as adult worker bees age, their IT shifts away from a diet relatively high in EAAs towards a diet that has 5 times less EAAs.

**Fig. 4:**
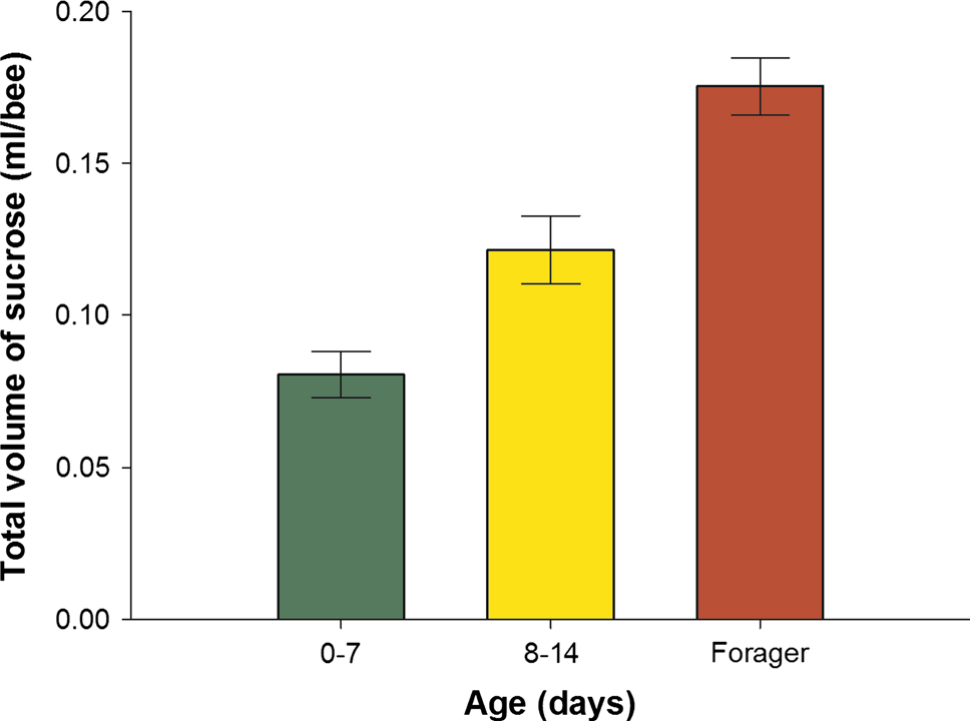
Dietary demand for carbohydrates increases with age (*n* = 20). Bees aged 0–7 days consumed the least amount of 1.0 M sucrose solution (pairwise LSD: vs. 8–14 days, *P* = 0.002; vs. foragers: *P* < 0.001); foragers consumed the most (8–14 day-old bees vs. foragers, pairwise LSD, *P* < 0.001)

This change in the need for dietary EAAs was also accompanied by a shift towards a greater demand for carbohydrates (Fig. 4). In a separate experiment, we found that foragers ate 60 % more 1.0 M sucrose solution than young bees (Fig. 4, GLZM: age, *χ*_2_^2^ = 50.3, *P* < 0.001). Thus, the shift in the IT with changes in caste reflected not only a reduced demand for EAAs, but also an increase in the overall amount of carbohydrates needed by foragers.

### Diets high in amino acid concentration result in poor longevity

**Fig. 5:**
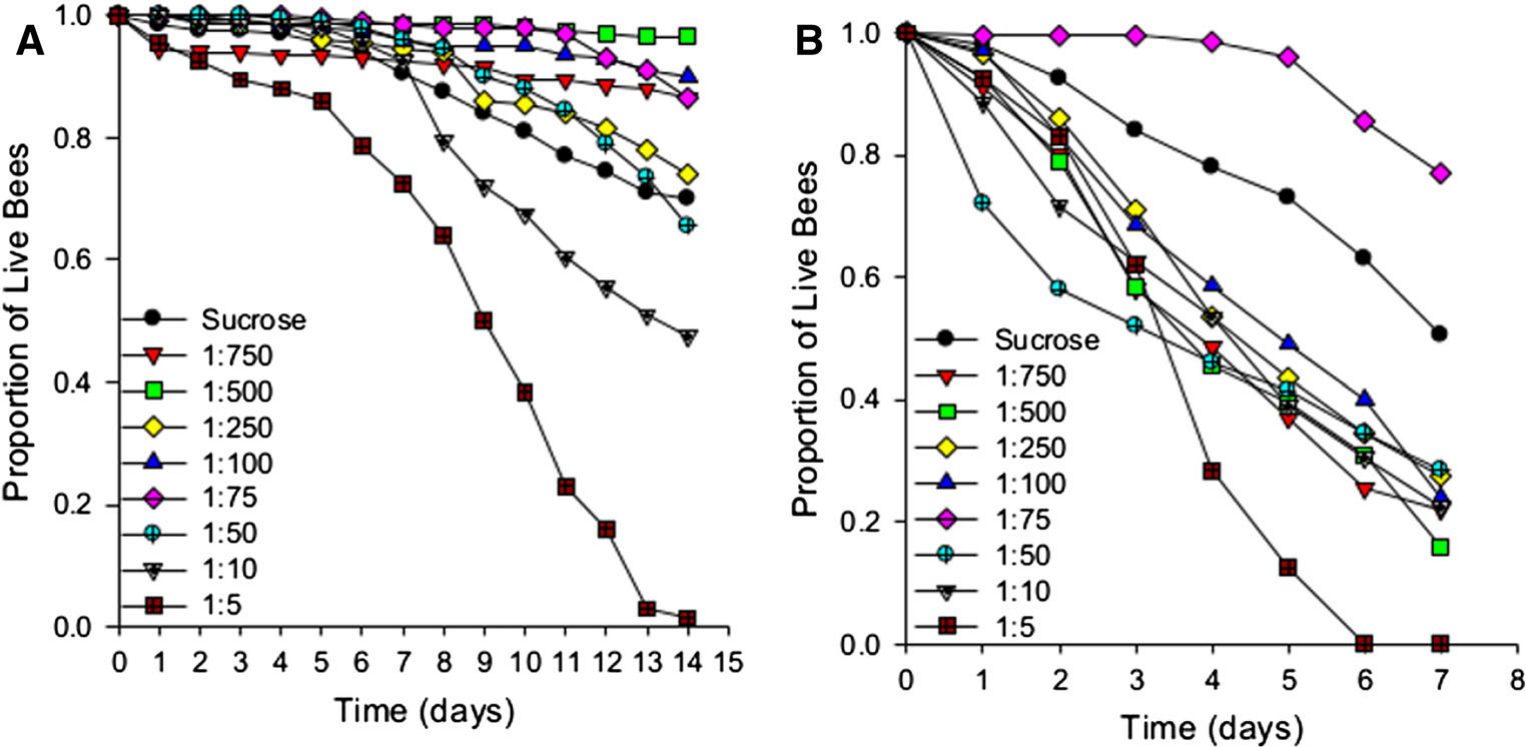
Survival of young bees and foragers is compromised by high concentrations of EAA. Survival on the amino acid diets for nurses over 14 days **a** and foragers **b** over 7 days. Each rail is represented as the total proportion of live bees/day (*n* = 10 cohorts of 20 bees each per rail). **a** Young bees fed on diets high in EAAs died at a faster rate than those on diets low in EAAs or sucrose alone (GLZM, Preg, two-way interaction, diet × day, *χ*_73_^2^ = 3.5 × 10^14^, *P* < 0.001). **b** Foragers fed diets containing EAAs also died at a faster rate (GLZM, Preg, two-way interaction, diet × day, *χ*_56_^2^ = 752.45, *P* < 0.001)

To identify costs associated with over consumption, we also compared the survival of each behavioural caste of the bees when fed each diet. Both young bees and foragers exhibited shorter lifespans on diets high in EAAs (Fig. 5). For the first 7 days, newly emerged bees had a lower proportional hazard of dying regardless of diet (Fig. 5a, Coxreg, days 0–7, sucrose × all other diets, HR = all <0.9 [95 % CI (confidence interval) 0.366–2.403], all *P* = 1.000). For bees aged 8–14 days, however, the risk of dying on diets high in EAAs increased dramatically such that there was a 6.5-fold increased risk when they were fed the 1:5 diet compared to sucrose (Coxreg, days 8–14, sucrose × 1:5 diet, HR = 6.065 [95 % CI 2.402–15.312], *P* < 0.001).

The proportional hazard of dying for foragers was also greater when they consumed diets containing EAAs (Fig. 5b, Coxreg, sucrose × 1:750–1:75, HR = 1–2.333 [95 % CI 0.202–9.03], *P* = 0.220). Their risk was ~3-fold greater when they were fed the 1:10 diet, and 8.3-fold greater when they were fed the 1:5 diet compared to sucrose (Coxreg, 1:10 × sucrose, HR = 3.00 [95 % CI 0.812–11.1], *P* = 0.067; 1:5 × sucrose, HR = 8.333, [95 % CI 2.52–27.6], *P* = 0.001).

### Young bees tolerate diets high in EAAs longer when exposed to QMP

**Fig. 6:**
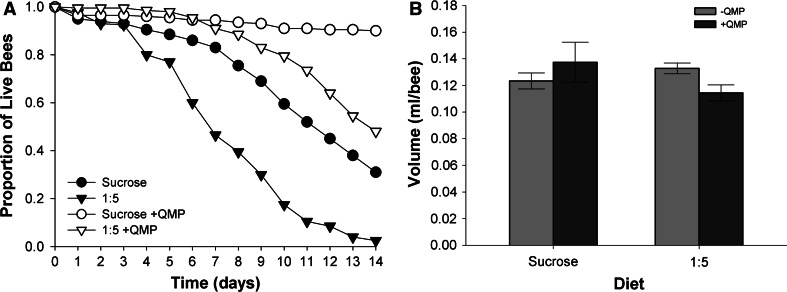
QMP exposure increases tolerance for dietary EAA. **a** Bees exposed to QMP have a higher rate of survival than same-aged queenless bees when fed a diet high in EAAs. Cohorts of 20 bees were confined to either sucrose or a 1:5 diet of EAAs and sucrose for 14 days. In the absence of QMP, young bees die faster when fed the 1:5 diet indicating that QMP is required to maintain them in the nurse-caste nutritional physiology. **b** Daily average consumption was not significantly different between bees exposed to QMP and bees that were not exposed to QMP. *n* = 10 cohorts per treatment

To identify whether EAAs affected survival of bees regardless of caste, we maintained bees in the ‘nurse-like’ caste using QMP and measured their rate of survival when fed a diet high in EAAs (1:5) or sucrose alone (Fig. 6a). Bees exposed to QMP had lower proportional hazard of dying over the 14-day period, regardless of diet (Coxreg, +QMP vs −QMP, HR = 0.688 [95 % CI 1.01–1.71], *P* = 0.045). As we observed in Fig. 4, when fed the 1:5 diet, bees had a greater risk of dying if they were reared in the absence of QMP (Coxreg, sucrose vs 1:5, HR = 6.50 [95 % CI 2.75–15.4], *P* < 0.001). When bees were exposed to QMP, the diet high in EAA (1:5) did not increase their risk of death during days 0–10 (Fig. 6a, Coxreg, sucrose vs 1:5, HR = 1 [95 % CI 0.791–1.27], *P* = 1.00). However, in days 11–14, regardless of QMP exposure, the 1:5 diet reduced survival compared to sucrose only (Coxreg, sucrose vs. 1:5, HR = 1 [95 % CI 1.932–11.27], *P* = 0.001). To verify this result, we also tested whether these curves were different fitting a Kaplan–Meier model; comparison of these treatments revealed that QMP-exposed bees fed the 1:5 diet died at a faster rate in the second week of the experiment (Kaplan–Meier, log-rank pairwise comparison, sucrose × 1:5, *χ*_1_^2^ = 23.9, *P* < 0.001). Bees exposed to QMP did not consume significantly more of either the sucrose or the 1:5 diets than the unexposed bees (Fig. 6b, 2-way GLZM: QMP trt × diet, *χ*_1_^2^ = 3.58, *P* = 0.058).

## Discussion

Our data demonstrate four important findings: (1) adult worker honeybees prioritize their dietary intake of carbohydrates over EAAs; (2) nutritional requirements for EAAs are greater for young bees, but shift as they age and become foragers; (3) workers show reduced survival on diets high in EAAs, but no cost is associated with eating diets composed only of carbohydrates; and (4) the risk of death associated with consumption of diets high in EAAs is a function of age and caste.

We expect that the IT for young bees (EAA:C of 1:50) mainly represents the IT required for somatic maintenance without the demands of producing glandular secretions or wax. Honeybees are unusual among insects, because adult workers have a specialisation that allows them to produce glandular secretions (e.g. royal jelly) as a form of care for offspring and other nest mates. Like mammalian milk, the glandular secretions produced by adult nurse bees are composed of proteins, fatty acids, and carbohydrates (Garcia-Amoedo and de Almeida-Muradian 2007; Kanbur et al. 2009; Peixoto et al. 2009) and are likely to place great nutritional demands on nurse bees during their production. In our experiments, young bees probably produced much less of their glandular secretions because they were not caring for larvae or exposed to brood pheromone. In addition to the demand for nutrients to produce royal jelly, young bees also sequester nutrients by making hexamerin proteins that are stored in the fat body and later used as a source of amino acids (Martins et al. 2008, 2010). In our experiments, the demand for EAA was high in broodless young bees, but perhaps not as great as if they had been in contact with brood or the queen in the colony. In this case, we predict that the young adult worker’s IT will be near the P:C ratio found in royal jelly (~1:1) (Garcia-Amoedo and de Almeida-Muradian 2007; Kanbur et al. 2009; Peixoto et al. 2009; Schmitzova et al. 1998).

In honeybees, behavioural caste is confounded with age: young bees are nurses, comb builders or guards and older bees are foragers (Robinson 1992). The transition to foraging is affected in part by proximity to the queen and exposure to QMP (Pankiw et al. 1998) as well as brood pheromone (Pankiw 2007). Workers that have little or no exposure to the queen or her pheromones undergo substantial physiological changes orchestrated by juvenile hormone (Robinson 1992) and begin to behave like foragers (Pankiw et al. 1998). Furthermore, in the absence of the queen, workers lose the ability to digest protein after their eighth day post-eclosion (Moritz and Crailsheim 1987). After the eighth day in our experiments with queenless workers, we also observed that the IT of our young bees shifted towards more carbohydrates. Using synthetic QMP as a tool to prevent young bees from transitioning to the forager caste, we were able to show that workers continue to tolerate high levels of dietary EAAs, even at levels exceeding their IT. In spite of having better survival early on, however, the QMP-exposed nurse bees fed with the 1:5 diet still died at a faster rate than those fed with sucrose in the final days of the experiment. It is possible that in this case, even though they were exposed to QMP, their lifespan was affected by the continued consumption of diets high in amino acids (Grandison et al. 2009) or that synthetic QMP alone was not sufficient to keep them in the ‘nurse-like’ caste (Maisonnasse et al. 2010).

The carbohydrate and lipid metabolism of bees is also affected by QMP (Fischer and Grozinger 2008). QMP maintains the abdominal fat of young bees and increases their resistance to starvation (Fischer and Grozinger 2008). Our data indicate that this resistance to starvation may be due to the fact that the presence of QMP maintains their physiological state such that they require much less carbohydrate. Thermal stress is another factor that could influence the within-hive bees’ demand for carbohydrates. In the presence of brood, young bees keep the brood warm using their flight muscles to produce heat when the temperatures outside of the colony drop (Simpson 1961; Fahrenholz et al. 1992; Stabentheiner et al. 2010). Our bees were neither exposed to brood nor did we investigate the influence of temperature on the IT, but we predict that young, within-hive bees performing endothermy would exhibit a greater demand for dietary carbohydrates. Based on our data, we also estimate that the workers’ demands for carbohydrate increase from 2 to 5 times when they become foragers. Foraging honeybees fly to and from the nest to collect food and water for the colony: an activity with high demands for energy (Suarez and Darveau 2005; Suarez et al. 2005). Indeed, the mass-specific metabolic rate of flying foragers is the highest of any animal recorded (Suarez et al. 1996). If placed under the demands of flight and carrying pollen loads, the energetic demands of foragers increase to +50-fold greater than at rest (Joos et al. 1997). For this reason, we predict the IT of flying foragers to be skewed even further towards the intake of carbohydrates than we were able to measure in our cohorts of bees confined to cages.

Previous studies of worker honeybee and ant nutrition also showed that workers die at a faster rate when they are forced to consume diets high in protein (Pirk et al. 2010; Dussutour and Simpson 2012). In our experiments, the foragers fed diets high in EAAs (1:10 and 1:5 diets) had much reduced survival because they were required to over-ingest EAA to obtain sufficient carbohydrates. Their premature mortality was not a function of a refusal to eat diets high in dietary EAA, indicating that consumption of the EAA was the cause of mortality. Even bees exposed to QMP and fed the 1:5 diet for 14 days had greater mortality during days 11–14 than those fed sucrose alone. The fact that honeybee workers are more likely to die as a function of eating EAAs may indicate that bees are not efficient at converting EAAs into energy via gluconeogenesis. Diets high in EAAs could cause metabolic stress and require that excess is excreted. Locusts fed diets high in protein excrete higher concentrations of amino acids in their faeces than those fed diets low in protein (Zanotto et al. 1994). In the cohorts fed diets high in EAAs, we also observed more defecation within the boxes. This could indicate that bees accumulated waste associated with excess amino acids or uric acid and had difficulty eliminating it.

A previous study showed that the IT of isolated cohorts of young worker honeybees was 1:12 (wt/wt) when they were fed solid diets based on proteins such as casein (Altaye et al. 2010). This is an almost 10-fold lower IT for EAAs compared to the IT of our young bees (~1:115 wt/wt, Table S1). The digestion of protein requires the production of proteases, which could place a greater demand on bees for EAAs (Moritz and Crailsheim 1987). Furthermore, proteins such as casein are composed of unequal ratios of amino acids when they are digested (Moritz and Crailsheim 1987; Szolderits and Crailsheim 1993), whereas our liquid diets were composed of all the EAAs at the same concentration. While we did not specifically test this, the fact that our IT was strongly skewed towards carbohydrates in comparison to an IT calculated for solid proteins (Altaye et al. 2010) implies that the ratios of EAAs in diet strongly affect dietary regulation of protein/EAA intake. This could be a general feature of protein regulation in animals, but has yet to be tested. In support of this, Altaye et al. (2010) found that diets rich in digestible protein such as royal jelly yielded a ratio skewed more towards carbohydrate (royal jelly: 1:14), whereas artificial diets that are likely to be more difficult for bees to digest predicted an IT of 1:11.

Previous studies have found that starvation and social isolation induce the transition to the foraging caste (Marco Antonio et al. 2008; Pankiw et al. 1998). The young worker bees in our study were not socially isolated and were given *ad libitum* access to food sources, yet they still continued on a trajectory towards a nutritional IT biased towards carbohydrate––consistent with them undergoing physiological changes that accompany foraging. Our data demonstrate that caste determines dietary needs of workers in the honeybee colony. Worker honeybees fail to survive if they do not consume sufficient dietary carbohydrates, and will over-ingest EAAs to obtain carbohydrate, even if it reduces their long-term survival. The influence of the queen, combined with feedback about the amount of food in the colony (Marco Antonio et al. 2008) and the presence of other foragers (Leoncini et al. 2004), is all likely to be the factors that affect the worker bee’s caste and hence its nutritional optimum.

## Electronic supplementary material

Below is the link to the electronic supplementary material.

Supplementary material 1 (DOCX 16 kb)
