# Supplementary material for: Nutritional balance of essential amino acids and carbohydrates of the adult worker honeybee depends on age
Source: Amino Acids. 2014 Mar 13;46(6):1449–58. doi: 10.1007/s00726-014-1706-2 (PMC4021167; doi:10.1007/s00726-014-1706-2)
Supplement: Supplementary file 1 — Supplementary material 1 (DOCX 16 kb) [file 726_2014_1706_MOESM1_ESM.docx]

| **Table S1. Dietary amino acid concentrations** | | | | | | | | |
| --- | --- | --- | --- | --- | --- | --- | --- | --- |
| **Diet** | **P:C (w/w)** | | | **Total AA (M)** | | **Individual AA (M)** | | |
| 1:750 | 1:1530 | | | 0.0013 | | 0.00013 | | |
| 1:500 | 1:1147 | | | 0.002 | | 0.0002 | | |
| 1:250 | 1:573 | | | 0.004 | | 0.0004 | | |
| 1:100 | 1:230 | | | 0.01 | | 0.001 | | |
| 1:75 | 1:153 | | | 0.013 | | 0.0013 | | |
| 1:50 | 1:115 | | | 0.02 | | 0.002 | | |
| 1:10 | 1:23 | | | 0.1 | | 0.01 | | |
| 1:5 | 1:11 | | | 0.2 | | 0.02 | | |
| **Note:** All amino acids diluted in 1M sucrose solution (342g/L) | | | | | | | | |
| **Table S2 Evaporation rates for EAA diets** | | | | |  | |  |  |
| ***Diet*** | | | ***Mean Evaporation*** | | ***Δ Concentration*** | | |  |
|  | | | **(mg/day)** | | **(%)** | | |  |
| Sucrose | |  | 0.178 | | 1.2 | | |  |
| 1:750 | |  | 0.330 | | 3.29 | | |  |
| 1:500 | |  | 0.160 | | 1.58 | | |  |
| 1:250 | |  | 0.165 | | 1.61 | | |  |
| 1:100 | |  | 0.166 | | 1.56 | | |  |
| 1:75 | |  | 0.350 | | 3.37 | | |  |
| 1:50 | |  | 0.350 | | 3.3 | | |  |
| 1:10 | |  | 0.179 | | 0.79 | | |  |
| 1:5 | |  | 0.243 | | 0.43 | | |  |
| ***Note:*** Mean evaporation is calculated across 5 consecutive days | | | | | | |  |  |
